# Physical Examination and Diagnostic Anatomy

**Published:** 1910-11

**Authors:** 


**Physical Examination and Diagnostic Anatomy.** By **Charles B. Slade,** M.D., Instructor in Physical Diagnosis, University and Bellevue Hospital Medical College, New York. 12 mo, of 146 pages. Illustrated. Philadelphia and London: W. B. Saunders Company. 1910. (Cloth, $1.25 net.)

This little book is designed to meet the needs of the student in the study of clinical anatomy. It very well describes the technic of physical examination. The illustrations are good, a large number of them having been drawn by the hand of the author. The subject matter is arranged to accord with what the writer believes to be the natural course the average student prefers to pursue in acquiring diagnostic knowledge. J. A. R.

